# Research on Deformation Prediction of VMD-GRU Deep Foundation Pit Based on PSO Optimization Parameters

**DOI:** 10.3390/ma17102198

**Published:** 2024-05-08

**Authors:** Ronggui Liu, Qing Zhang, Feifei Jiang, Juan Zhou, Jianxia He, Zhongyang Mao

**Affiliations:** 1 Faculty of Civil Engineering and Mechanics, Jiangsu University, Zhenjiang 212013, China; 2 China State Construction Engineering (Macau) Co., Ltd., Macau 999078, China; zhangqing1@cohl.com (Q.Z.); hejianxia@cohl.com (J.H.); 3 School of Civil Engineering, Nantong Institute of Technology, Nantong 226000, China; zhoujuan198808@163.com; 4 College of Materials Science and Engineering, Nanjing Tech University, Nanjing 211800, China; mzy@njtech.edu.cn

**Keywords:** deformation prediction of deep foundation pit, particle swarm optimization algorithm, variational mode decomposition, gated recurrent unit

## Abstract

As a key guarantee and cornerstone of building quality, the importance of deformation prediction for deep foundation pits cannot be ignored. However, the deformation data of deep foundation pits have the characteristics of nonlinearity and instability, which will increase the difficulty of deformation prediction. In response to this characteristic and the difficulty of traditional deformation prediction methods to excavate the correlation between data of different time spans, the advantages of variational mode decomposition (VMD) in processing non-stationary series and a gated cycle unit (GRU) in processing complex time series data are considered. A predictive model combining particle swarm optimization (PSO), variational mode decomposition, and a gated cyclic unit is proposed. Firstly, the VMD optimized by the PSO algorithm was used to decompose the original data and obtain the Internet Message Format (IMF). Secondly, the GRU model optimized by PSO was used to predict each IMF. Finally, the predicted value of each component was summed with equal weight to obtain the final predicted value. The case study results show that the average absolute errors of the PSO-GRU prediction model on the original sequence, EMD decomposition, and VMD decomposition data are 0.502 mm, 0.462 mm, and 0.127 mm, respectively. Compared with the prediction mean square errors of the LSTM, GRU, and PSO-LSTM prediction models, the PSO-GRU on the PTB0 data of VMD decomposition decreased by 62.76%, 75.99%, and 53.14%, respectively. The PTB04 data decreased by 70%, 85.17%, and 69.36%, respectively. In addition, compared to the PSO-LSTM model, it decreased by 8.57% in terms of the model time. When the prediction step size increased from three stages to five stages, the mean errors of the four prediction models on the original data, EMD decomposed data, and VMD decomposed data increased by 28.17%, 3.44%, and 14.24%, respectively. The data decomposed by VMD are more conducive to model prediction and can effectively improve the accuracy of model prediction. An increase in the prediction step size will reduce the accuracy of the deformation prediction. The PSO-VMD-GRU model constructed has the advantages of reliable accuracy and a wide application range, and can effectively guide the construction of foundation pit engineering.

## 1. Introduction

With the rapid development of the economy, the pace of urban construction has gradually accelerated, and much urban construction focuses on underground engineering, and more and more underground projects such as subway projects, underground corridors, and underground commercial buildings have appeared. In the process of foundation pit excavation, on the one hand, with the increase in the excavation depth, the deformation data of the foundation pit present non-stationary and nonlinear deformation laws, and the deformation laws of different sides of the same foundation pit are also different. On the other hand, because the deep foundation pit is located in the central area of the city and there are many building groups, the influence on the surrounding buildings should be taken into account in the excavation process, and the deformation of the foundation pit should be controlled dynamically.

At present, the deformation control of a foundation pit is based on the real-time monitoring of the monitoring points, obtaining and analyzing the monitored data, and comparing them with the deformation safety threshold set in the design. If the deformation data of the foundation pit exceed the set threshold, measures should be taken to protect it to avoid causing foundation pit accidents and unnecessary personnel and property losses. However, the deformation of a foundation pit is affected by many factors such as geological conditions, the surrounding environment, the support scheme, and the excavation form, so it is worth exploring and solving to accurately predict the deformation of the foundation pit. In the field of foundation pit deformation, generally, traditional methods rely on design schemes and soil exploration reports, soil physical and mechanical parameter values, and structural material parameters. Specifically, soil distribution, data related to soil physical and mechanical properties, depth, width, the support structure form (such as pile walls, soil nail walls, etc.), and parameters (such as pile diameter, pile length, spacing, etc.) of deep foundation pits. However, the traditional finite element analysis has certain limitations. The finite element method carries out mechanical simulations based on the preliminary survey data, but the actual construction stage is far more complicated than the simulation, and the deformation results obtained cannot be accurately mapped to a certain point in the future. With the development of machine learning, it has been applied more and more widely in different engineering fields [1], among which deep learning has achieved good application effects in many fields, providing a new idea for deep foundation pit deformation prediction. Compared with the shortcomings of traditional finite element analysis, linear regression, the support vector machine, the BP neural network, and other prediction methods are all static models. Although they can fit data to a certain extent, they have poor performance for deformation data with non-stationary, non-linear, and time-dependent characteristics [2]. In deep learning, the traditional Recurrent Neural Network (RNN) model is more suitable for data with time-dependent characteristics, and it can learn the internal change law of the time series [3,4], but it has problems of gradient disappearance and gradient explosion in practical applications [5]. Hochreiter et al. [4] proposed the Long Short-Term Memory (LSTM) network to solve this problem. Then CHO et al. [6] adjusted the three gate structures of the LSTM network and proposed a Gated Recurrent Unit (GRU) network consisting of an update gate and reset gate.

Based on the above, many scholars have carried out a lot of research on the prediction of non-stationary and nonlinear data and time series data. In order to predict the surface settlement caused by underground mining activities, Sepehri et al. [7] established a three-dimensional finite element model, and the average relative error between the effect and the measured data was 7.95%, which was in good agreement with the actual situation. Xianglong Luo et al. [8] introduced Empirical Mode Decomposition (EMD) to process the non-stationary data in view of the non-stationary and nonlinear characteristics of the structural deformation data. Shaoyi Yang et al. [9], in order to reduce the instability of short-term wind speed data and further improve the model prediction accuracy, processed historical data by Variational Mode Decomposition (VMD) and then further improved the prediction accuracy. Aiming at short-term passenger flow data with nonlinear and non-stationary characteristics, Liang D et al. [10] adopted a VMD-LSTM combined prediction model to process and forecast the data and achieved good results. Hailin Li et al. [11] optimized the LSTM prediction model by setting different algorithm strategies on the obtained monitoring data, and came to the conclusion that the Adam optimization algorithm model had the highest prediction accuracy. Considering the advantages and disadvantages of the GM(1,1) model and BP neural network, Dongge Cui et al. [12] first used PSO-GM(1,1) to predict and extract the trend item of deformation data, and then used the PSO-BP network to correct the residual sequence and superposition to obtain the predicted value of the PSO-GM-BP model. Q Liu et al. [13] used the combined model of the wavelet transform and BP neural network to predict the deformation of a deep foundation pit. Compared with the prediction results of the original data, the relative error of the data after wavelet transform was reduced, which verified that the processed data were more conducive to the improvement of the model prediction effect. Jing Chuankui et al. [14] predicted the settlement data of three foundation pits by different optimization algorithms combined with the Exponential Power Product Model and obtained good model effects. ZHANG et al. [3] used the LSTM algorithm, which is more suitable for time series, to predict the horizontal deformation data of a foundation pit. Compared with the BP neural network and gray prediction model, LSTM has a better prediction performance. According to the different excavation stages, a phased prediction was made, and a similar model performance was obtained. Ma Qingwen et al. [15] proposed the EWT-NARX prediction model by combining the empirical wavelet transform (EWT) with the Nonlinear Auto-Regressive model with Exogenous Inputs (NARX). The results show that compared with the EMD-NAR, EWT-NAR, and NARX models, the proposed model has higher accuracy.

To sum up, the instability and nonlinearity of data will cause great obstacles to prediction. Different scholars process nonlinear and unstable data through various decomposition methods in order to achieve better model effects. The deformation data of a deep foundation pit also have their own characteristics, and it is difficult to obtain a good prediction effect by a direct prediction, so VMD is introduced to process the original data. As the VMD algorithm needs to set parameters subjectively, the PSO algorithm is introduced to optimize VMD parameters. Considering that the characteristics of deep foundation pit deformation data are highly dependent on time, LSTM and GRU, as variants of RNN, are very suitable for processing time series data.

At present, there are few pieces of research in the field of deep foundation pit deformation predictions based on the GRU network. LSTM and GRU models are used to make short-term and long-term predictions of deformation data, in order to achieve a better prediction effect, so as to scientifically and safely protect deep foundation pit engineering. With reference to the final prediction results, appropriate measures are taken in time to avoid risks and ensure the safety and stability of the whole construction process. The contributions of this paper are as follows:

(1) Propose an LSTM model optimized based on the PSO algorithm to predict deep excavation deformation, and select hyperparameters of the LSTM network through the PSO algorithm to avoid the limitations of manual parameter tuning.
(2) Propose to use VMD optimized by the PSO algorithm to process the deformation data of deep foundation pits, so that non-stationary and unstable data can be better utilized by the model. Introduce the PSO algorithm to optimize the K value of VMD and obtain the decomposition number. In addition, the correctness of the decomposition number obtained by energy difference verification was added, and the EMD decomposition method was also added as a comparison. After VMD processing, the phenomenon of mode mixing in the data was significantly reduced.
(3) Experiment with setting different prediction steps for the prediction model to meet the application scenario requirements in different practical situations. The root mean square error was used as an indicator to evaluate the performance of the prediction model, and the results showed that increasing the prediction step size would increase the prediction error.
(4) In order to verify the effectiveness of the proposed model, it was compared with different models, such as traditional LSTM, GRU, and PSO-LSTM. The mean square error of different models was calculated for model performance evaluation, and the results showed that the model can make a better prediction performance.

The remaining parts of this article are organized as follows: Section 2 describes the methods used to build the model and proposes a VMD-GRU prediction method based on PSO optimization, Section 3 applies the constructed model to a case study and analyzes it, and finally, conclusions are drawn in Section 4.

## 2. Materials and Methods

### 2.1. Particle Swarm Optimization

Particle swarm optimization (PSO) is an evolutionary computing technique. The particles in the algorithm constantly update their position and velocity attributes according to the individual local extremum and the global extremum of the population [16,17]. The size, shape, and composition of particles are not the key attributes that algorithms focus on, that is, particles are abstracted as massless entities in the parameter space to be optimized, and their key attributes are position and velocity. Position: represents the current position of the particle in the search space, which can be understood as the potential solution to the problem. Velocity: determines the direction and step size of particle movement in search space, used to update particle position.

If a population $X=\left(X_{1},X_{2},\ldots ,X_{M}\right)$ exists in a D-dimensional space, then the position $S_{i}$ and the velocity $V_{i}$ of the i particle in space can be expressed as

(1) $$\left\{ \begin{array}{l} S_{i}=\left(S_{i1},S_{i2},\ldots ,S_{iD}\right)\\ V_{i}=\left(V_{i1},V_{i2},\ldots ,V_{iD}\right) \end{array}\right.$$

The individual local extreme value $p_{i}$ and the population total extreme value G can be expressed as

(2) $$\left\{ \begin{array}{l} p_{i}=\left(p_{i1},p_{i2},\ldots ,p_{iD}\right)\\ G=\left(G_{1},G_{2},\ldots ,G_{D}\right) \end{array}\right.$$

The position $S_{i}$ and velocity $V_{i}$ of each particle are iteratively updated according to the local extremum $p_{i}$ of the individual and the total extremum G of the population. The iterative updates of velocity and position can be expressed as

(3) $$\left\{ \begin{array}{l} v_{id}^{k+1}=\omega v_{id}^{k}+c_{1}\eta (P_{id}^{k}-s_{id}^{k})+c_{2}\eta (G_{d}^{k}-s_{id}^{k})\\ s_{id}^{k+1}=s_{id}^{k}+v_{id}^{k+1} \end{array}\right.$$

where i=0,1,…; k is the number of iterations and $v_{in}^{k+1}$ is the velocity of particle i in the k+1 iteration of n. The value ranges of inertia weights ω and η are [0, 1], and $c_{1}$ and $c_{2}$ are learning factors. $P_{in}^{k}$ is the position of particle i at the individual extreme point of the n dimension in the k iteration. $G_{n}^{k}$ is the position of the global value point of the particle swarm population in the n dimension in the k subiteration. When the particle swarm algorithm initializes parameter settings, it will provide a random particle (which represents a random solution to the problem), including basic position and velocity information.

### 2.2. Variational Mode Decomposition

Variational mode decomposition is a non-recursive and adaptive signal processing method proposed by D et al. in 2014 [18]. It has a good anti-noise ability and can overcome the frequency aliasing problem caused by EMD decomposition.

In this method, the time series data are decomposed into K mode $u_{k}(t)$ by iteratively searching the optimal solution of variational modes. If the original signal f is decomposed into K IMF components, the corresponding constraint variational model is expressed as

(4) $$\left\{ \begin{array}{l} \underset{\left\{u_{k}\right\},\left\{w_{k}\right\}}{\min}\left\{\sum_{k}\|\partial_{t}\left[\left(\delta (t)+\frac{j}{\pi t})u(t\right)\right]e^{-jw_{k}t}{\|}_{2}^{2}\right\}\\ s.t\;\sum_{k}uk=f \end{array}\right.$$

where f is the input signal; $u_{k}$ is the k modal components obtained after decomposition; δ(t) is the pulse function; and $w_{k}$ is the center frequency of each modal component.

The augmented Lagrange function is introduced to solve the above variational problem, expressed as

(5) $$\begin{array}{l} L(\left\{uk\right\},\left\{wk\right\},\lambda )=\alpha \sum_{k}\|\left[(\partial_{t}+\frac{j}{\pi t})u(t)\right]e^{-jw_{k}t}{\|}_{2}^{2}+\\ \|f(t)-\sum_{k}u(k)(t){\|}_{2}^{2}+\langle \lambda (t),f(t)-\sum_{k}u_{k(t)}\rangle \end{array}$$

where α is the penalty parameter and λ is the Lagrange multiplier. The alternating direction multiplier algorithm is used to iteratively find the optimal solution, and then the signal is decomposed into different modal components $u_{k}$.

(6) $$\left\{ \begin{array}{l} u_{k}^{n+1}(\omega )=\frac{f(\omega )-\sum_{i\neq k}u_{i}(\omega )+\frac{\lambda (\omega )}{2}}{1+2\alpha {\left(\omega -\omega_{k}^{n}\right)}^{2}}\\ \omega_{k}^{n+1}=\frac{\int_{0}^{\infty }\omega {\left|u_{k}^{n+1}(\omega )\right|}^{2}d\omega }{\int_{0}^{\infty }{\left|u_{k}^{n+1}(\omega )\right|}^{2}d\omega }\\ \lambda^{n+1}(\omega )=\lambda^{n}(\omega )+\rho \left(f(\omega )-\sum_{k}u_{k}^{n+1}(\omega )\right) \end{array}\right.$$

where ρ is the noise tolerance and $u_{k}^{n+1}(\omega )$, $u_{i}(\omega )$, f(ω), and λ(ω) becomes $u_{k}^{n+1}(t)$, $u_{i}(t)$, f(t), and λ(t) after the Fourier change.

After repeating the above steps until the set error ε is satisfied, the iteration termination condition is expressed as

(7) $$\sum_{k}\|u_{k}^{n+1}-u_{k}^{n}{\|}_{2}^{2}/\|u_{k}^{n}{\|}_{2}^{2}<\varepsilon$$

**Figure 1:**
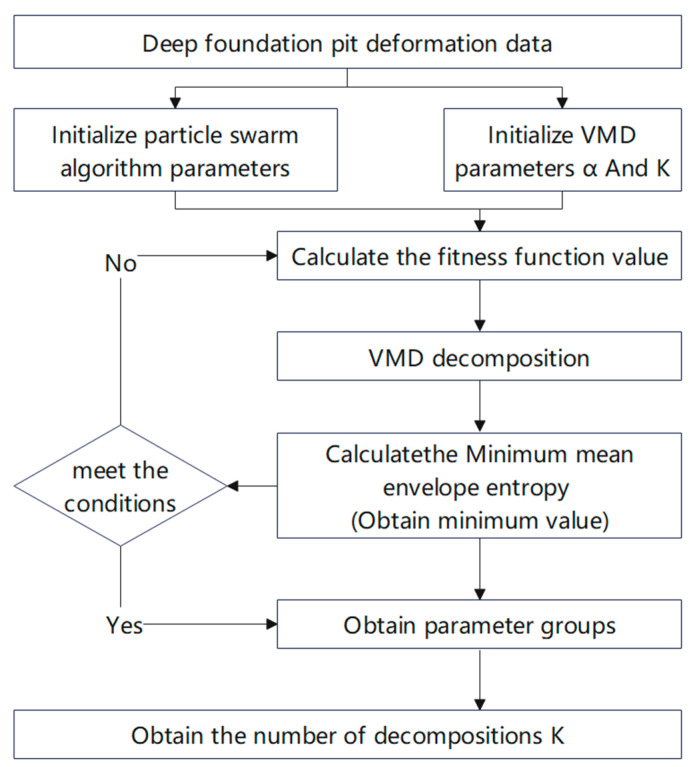
PSO-VMD flow chart.

The VMD algorithm is a signal decomposition method based on frequency domain decomposition, and its basic steps are as follows. The flow chart is shown in Figure 1.

(1) Initialize the decomposition signal, (n=0);
(2) Start the loop (n=n+1);
(3) Update, $u_{k}$, $\omega_{k}$, and λ in the loop according to Equation (6);
(4) According to Equation (7), determine whether the set error ε is satisfied. If it is not satisfied, return to step 2. If it is satisfied, the iteration is stopped.

### 2.3. PSO Optimizes VMD Parameters

Since the VMD algorithm needs to initialize many parameters before iteration [19], the key is to determine the decomposition mode number K. According to the optimal theory of VMD decomposition, the energy matched by the original signal should be consistent with the energy sum of each component, and the unreasonable setting of the decomposition number will lead to the mismatch between the component energy and the original energy. The energy difference method formula can be expressed as [20]

(8) $$E_{u}^{l}=\sqrt{\frac{\sum_{i=1}^{n}x_{l}^{2}(i)}{n}},l=1,2,\ldots ,u$$

(9) $$E_{u}=\sum_{l=1}^{u}E_{u}^{l}$$

(10) $$\theta_{u,u-1}=\frac{\left|E_{u}-E_{u-1}\right|}{E_{u}}$$

where u is the decomposition number; $x_{l}$ is the l component sequence in the modified resolution number; and $E_{u}$ is the sum of energies when the decomposition number is u. $\theta_{u,u-1}$ is the ratio of the energy difference and when it changes significantly, the signal is overdecomposed, and the best decomposition number is K=u−1.

The decomposition result of the VMD algorithm is not only affected by the decomposition number K, but also by the penalty parameter α. Generally, the experience value of α is 1.5 to 2 times the length of the sampling point. In order to ensure objectivity and avoid greater human interference, the particle swarm optimization algorithm is introduced to optimize the parameter group [α,K] in the VMD algorithm. After determining the parameter [α,K] to be optimized, an appropriate fitness function needs to be determined. This paper refers to the method in reference [21] and uses the Minimum mean envelope entropy (MMEE) as the fitness function for the PSO optimization of VMD. The MMEE and the corresponding parameter set can be expressed as

(11) $$\left\{ \begin{array}{l} \langle \hat{\alpha },\hat{K}\rangle =\underset{\left(\alpha ,K\right)}{\arg\;\min}\left\{\frac{1}{\hat{K}}\sum_{i=1}^{\hat{K}}H_{\mathrm{en}}(i)\right\}\\ H_{\mathrm{en}}(i)=-\sum_{i=1}^{K}p_{i}\log_{2}(p_{i}) \end{array}\right.$$

where $\langle \hat{\alpha },\hat{K}\rangle$ is the parameter group corresponding to the minimum average envelope entropy and $H_{\mathrm{en}}(i)$ is the envelope entropy of each mode $u_{k}$. In order to ensure comparability, it is necessary to normalize the envelope entropy $u_{k}$ of each mode, and $p_{i}$ is the normalized envelope value of each mode $u_{k}$.

### 2.4. Gated Recurrent Unit

**Figure 2:**
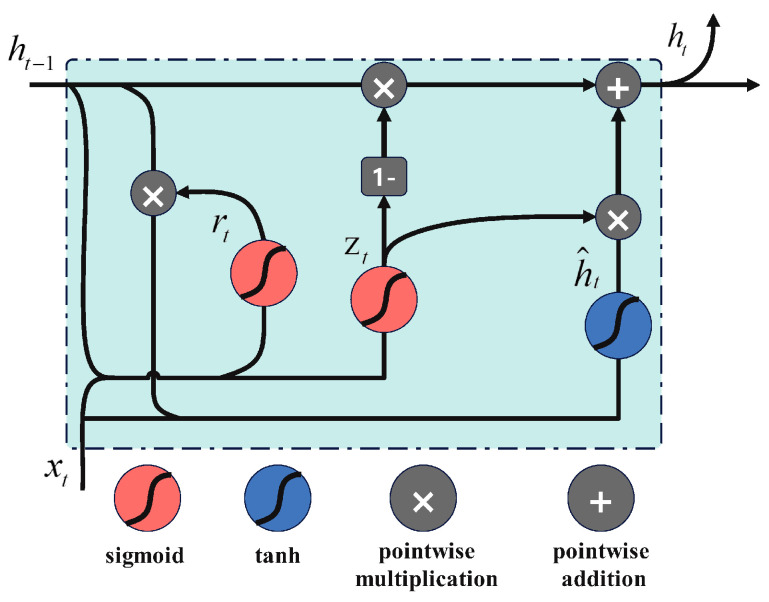
Cell structure of gated recurrent unit.

The GRU has a gating structure similar to that of LSTM, through which the flow of input information is controlled to better learn the dependence of larger time steps in the sequence. The cell structure of GRU is shown in Figure 2. Compared with LSTM, GRU has fewer parameters, faster convergence, and better performance in some small sample datasets [22].

It can be seen from Figure 2 that the update gate $z_{t}$ and reset gate $r_{t}$ in each GRU cell structure will select the input data at the current moment and then send it to the cell structure at the next moment. This closely connected structure is more suitable for data that are dependent on the current output of the sequence and the previous output.

The update gate, by concatenating the output $h_{t-1}$ of the previous time and the input $x_{t}$ of the current time (the current time input has different meanings in different works and it represents a numerical point in the time series used for deep excavation deformation training), then, through the sigmoid function, the update gate is responsible for controlling the influence of the state information of the previous moment on the current moment state. The larger the update gate value is, the more the state information of the previous moment is brought in; the smaller the update gate value is, the more information is forgotten. The reset gate, consistent with the updates, will first concatenate the output $h_{t-1}$ at the previous time and the input $x_{t}$ at the current time, and then perform numerical transformation through the sigmoid function. The reset gate is responsible for controlling the degree to which the status information of the previous moment is ignored. The smaller the reset gate value, the more the status information is ignored. The data through the reset gate are multiplied with the matrix composed of the output $h_{t-1}$ at the previous time and the input $x_{t}$ at the current time, and then the state ${\hat{h}}_{t}$ at this time is obtained by tanh function transformation. At this time, ${\hat{h}}_{t}$ is not the $h_{t}$ output by the cell structure, and $h_{t}$ is affected by ${\hat{h}}_{t}$, $z_{t}$, and $h_{t-1}$ parameters, which can be expressed as

(12) $$\left\{ \begin{array}{l} z_{t}=\sigma \left(W_{z}\cdot \left[h_{t-1},x_{t}\right]\right)\\ r_{t}=\sigma \left(W_{r}\cdot \left[h_{t-1},x_{t}\right]\right)\\ {\hat{h}}_{t}=\tanh\left(W\cdot \left[r_{t}\times h_{t-1},x_{t}\right]\right)\\ h_{t}=\left(1-z_{t}\right)\times h_{t-1}+z_{t}\times {\hat{h}}_{t} \end{array}\right.$$

In the formula, the value of $z_{t}$ can effectively control the retention of $h_{t-1}$ in the cell structure. In other words, the closer the value of $z_{t}$ is to 0, the information of $h_{t-1}$ can be well received by $h_{t}$ in the update. Studies show that, with appropriate parameter initialization, the recurrent neural network can also learn the long-term dependence relationship well [23], which alleviates the problem of gradient disappearance to a certain extent [24].

### 2.5. Particle Swarm Optimization of GRU Parameters

**Figure 3:**
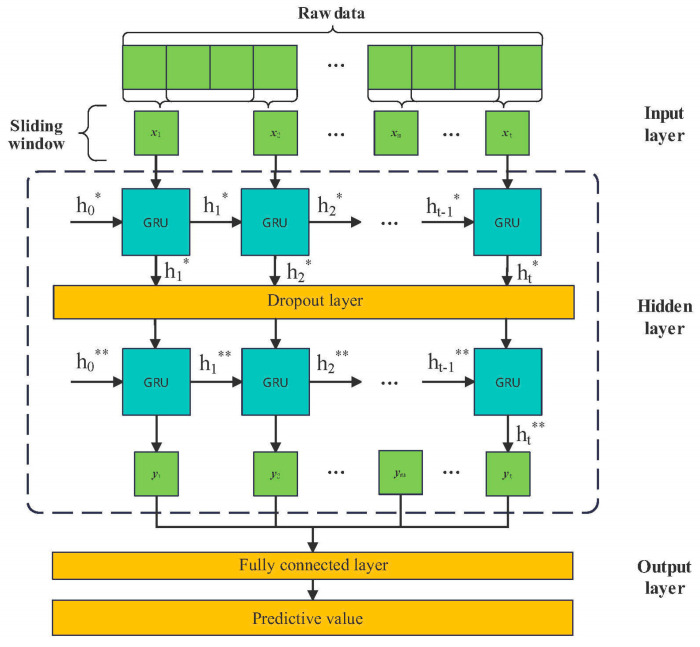
Double GRU layer network structure.

In deep learning, the efficiency and accuracy of the model are affected by the selection of hyperparameters. Generally, in the network structure, the hidden layer is usually composed of the simple stacking of cell structures, but the dependence of large time steps in the sequence is difficult to learn. By increasing the number of layers, the network can have stronger capabilities, but it will bring problems such as the overfitting of the model and too long a training time. The structure of the two-layer GRU network is shown in Figure 3.

It can be seen from the figure that the sliding window in the network is 3, that is, the data of every three periods in the data are taken as a set of data from the input layer to the GRU of the first layer and the calculation starts. The size of the sliding window determines the size of the time step t, where $h_{0}^{*},h_{1}^{*},\ldots$ represents the information transmission of the GRU of the first layer and $h_{0}^{**},h_{1}^{**},\ldots$ represents the information transmission of the GRU of the second layer. The Dropout layer is set within the adjacent GRU layer so that the backward units in the hidden layer do not learn, which is highly dependent on previous features, thus avoiding the overfitting of the model. $y_{1},y_{2},y_{3},\ldots ,y_{t}$ represents the output data of the neural network. $y_{t}$ is the predicted value of the output of the last GRU layer. In deep learning, the unreasonable setting of the Batch size may cause the model to fail to converge. If you want to achieve a better model effect, selecting the right hyperparameter is very important for the efficiency, structure, and accuracy of the model. In order to determine the optimal hyperparameters more reasonably and accurately, the minimum root-mean-square error is used as the fitness function of the PSO algorithm to optimize GRU parameters. The list and range of PSO-GRU optimization parameters are shown in Table 1.

Here, Num units are the vector dimensions’ output in the network and Num layers indicates the number of layers stacked by the GRU. When Num layers = 2, the GRU of the second layer calculates the output result of the first layer as the input signal of the second layer. In the constructed GRU model, only the dropout layer is added between layers. When the Dropout is not 0, the data transmitted between layers of the network will be discarded with a certain probability. Epochs is the number of iterations. If the Batch size parameter is small, it is easily disturbed by noise, so the loss function cannot converge. When the batch size parameter is large, the Epochs required will increase to achieve the same model effect. When constructing the model in the paper, the scope of PSO algorithm optimization is determined based on the size of the dataset in order to find a suitable hyperparameter combination for the current task and model.

The main idea used in particle swarm optimization to optimize the parameters of the GRU model is to take the parameters that need to be optimized as PSO particles, and give the range of this particle, which will help the algorithm converge. The five parameters are set in Table 1, which means that these particles will find the optimal parameter combination in a 5-dimensional space with the minimum fitness value as the standard.

### 2.6. PSO-VMD-GRU Model Prediction Process

**Figure 4:**
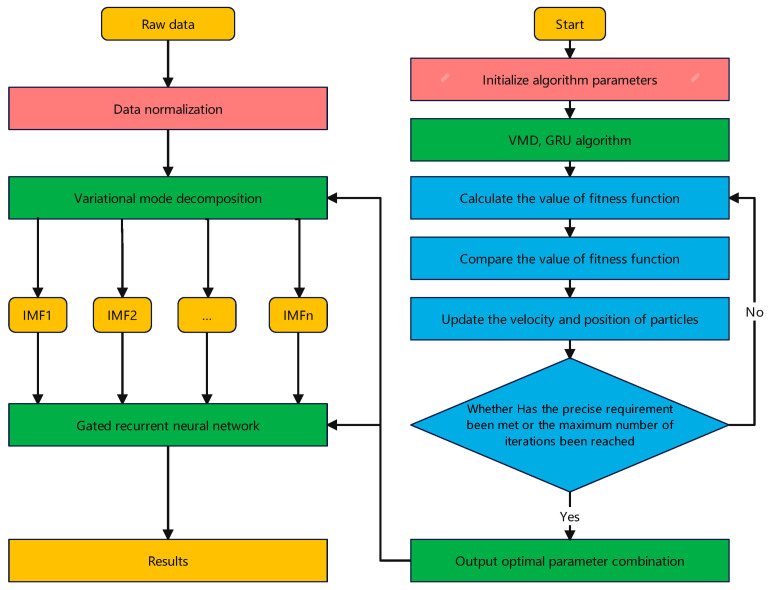
PSO-VMD-GRU prediction model process.

The particle swarm optimization algorithm is used as the parameter optimization algorithm of VMD and GRU, and the PSO-VMD-GRU deep foundation pit deformation prediction model is established. The working flow of the model is shown in Figure 4.

If the original time series data are set as f(t)(t=1,2,…,n), the steps to predict the deformation value of foundation pit engineering are as follows.

(1) The original time series data f(t) are normalized.
(2) K IMF are obtained by the VMD method which is optimized by the PSO algorithm.
(3) The K IMF training set and test set are divided.
(4) The PSO algorithm is used to optimize the hyperparameters of the GRU prediction model, and the optimized prediction model is obtained.
(5) The optimized prediction model is used to forecast the test set divided by IMF.
(6) The predicted value Y of foundation pit deformation is obtained by the summation of equal weights and superposition. The formula is as follows
  (13) $$Y=IMF_{1}+IMF_{2}+\ldots +IMF_{K}$$
  where Y is the predicted value of foundation pit deformation and $IMF_{i}$ is the predicted value of each intrinsic mode function, i=1,2,…,K.

### 2.7. Model Training

Before training the prediction model, it is necessary to divide the preprocessed data into training, validation, and testing sets. The parameters of the prediction model are adjusted using the training and validation sets, and the optimal parameter combination is selected to predict the test set. The data of deep excavation engineering have temporal characteristics. If general cross validation is used for set partitioning, the problem of the test set data appearing before the training set may occur. Therefore, this article assumes that the monitoring point data have *f*(*t*) periods, and the prediction step of the prediction model is *m* periods. The data used for training are processed according to a sliding window size of 3, that is, data from periods 1 to 3 are used as network inputs, and data from period 4 are used as model output target values. The data from period 2 to 4 should be taken as the network input, period 5 as the model output target value, and so on. The training set and validation set should be divided into 90% and 10% of *f*(*t*)-*m*-3 stages, respectively. The horizontal displacement PTB01 monitoring point has a total of 355 periods of data after interpolation processing, and 352 periods of data after sliding window processing. Therefore, the training set, validation set, and test set of the prediction model with a prediction step size of 5 for the PTB01 monitoring point are 312 periods, 35 periods, and 5 periods, respectively. The prediction model uses the Adam algorithm as the optimizer, the tan function as the activation function, and the mean square error loss as the loss function for training.

### 2.8. Model Effect Evaluation Index

In order to comprehensively evaluate the model effect, the Root Mean Square Error (RMSE) and the Mean Absolute Error (MAE) were used as the evaluation indexes of the model effect. The root-mean-square error $E_{rmse}$ is expressed as

(14) $$E_{rmse}=\sqrt{\frac{1}{N}{\sum_{t=1}^{N}\left(y_{t}-{\hat{y}}_{t}\right)}^{2}}$$

The mean absolute error $E_{mae}$ is expressed as

(15) $$E_{mae}=\frac{1}{N}\sum_{t=1}^{N}\left|y_{t}-{\hat{y}}_{t}\right|$$

where ${\hat{y}}_{t}$ is the deformation value predicted by the model for phase t; $y_{t}$ is the actual deformation value of phase t; and N is the total number of monitoring periods.

## 3. Simulation Verification and Results

### 3.1. Data Analysis

**Figure 5:**
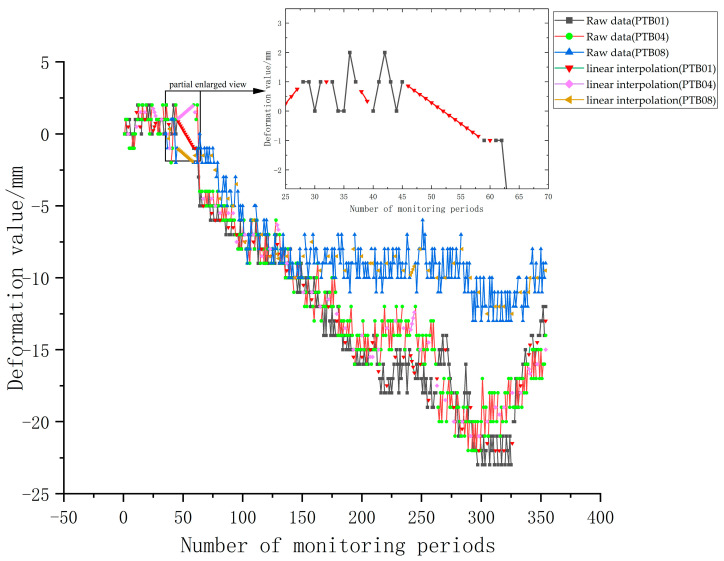
Raw data of monitoring points PTB01, PTB04, and PTB08.

In order to verify the feasibility and effectiveness of the proposed method, data from the east side (PTB01), north side (PTB04), and west side (PTB08) of a deep foundation pit project between 26 May 2022 and 15 May 2023 were selected (obtained by equipment such as level and total station, the data collection interval in the article is 1 day). Due to the influence of monitoring technology and on-site conditions, there are some missing values in the monitoring data. The linear interpolation method was used to process the missing values, as shown in Figure 5. The detailed information about the raw data is shown in Table 2.

The set coordinates $\left(t_{m},y_{n}\right)$ and $\left(t_{n},y_{n}\right)$ and the deformation value y corresponding to the number of monitoring periods t in the interval $\left[t_{m},t_{n}\right]$ can be expressed as

(16) $$y=y_{m}+\left(t-t_{m}\right)\frac{y_{n}-y_{m}}{t_{n}-t_{m}}$$

where $y_{m}$ is the deformation value corresponding to the time point where the number of monitoring periods is n, and $t_{m}$ is the time point where the number of monitoring periods is m.

Firstly, from the point of view of the maximum value of monitoring point data, due to the influence of surrounding conditions and excavation construction, there are certain differences in the maximum deformation value of different monitoring points. The maximum deformation value of the east and north sides of the foundation pit is −23 mm and −22 mm, respectively, and the maximum deformation value of the west side is −13 mm. Secondly, from the perspective of the data change trend, the three groups of data all show a large deformation amplitude and have the characteristics of being nonlinear and non-stable. Due to the construction near the monitoring points of PTB01 and PTB04, the deformation value continued to increase after stage 125, while the deformation data of the monitoring point of PTB08 on the west side of the foundation pit gradually stabilized. After considering the completeness of the deformation monitoring key points and point data, the PTB01 monitoring point adjacent to the public building foundation on the east side of the foundation pit and the PTB04 monitoring point adjacent to the residential building foundation on the north side of the foundation pit were selected as the research object. From the changes of the east and north monitoring points as a whole, the data were in a relatively stable state during the period from 1 to 50. From the 51–200 period, the data decreased significantly. From the 200th to 320th period, the data of the monitoring points showed a sharp decline after a slight rebound, accompanied by local floating. After 320, the data showed an obvious rebound trend. The rebound time of the PTB01 monitoring point lags behind that of the PTB04 monitoring point due to factors such as phased excavation and adjacent construction.

### 3.2. Deformation Sequence Decomposition

On the whole, the data of the three monitoring points show similar nonlinear and non-stationary changes. If the original time series data are used to construct the prediction model, the model will learn the unnecessary change features, which will affect the overall prediction effect of the model. Therefore, it is necessary to stabilize the original data to make it easier for the model to learn the law of the data change and improve the prediction effect of the model.

**Figure 6:**
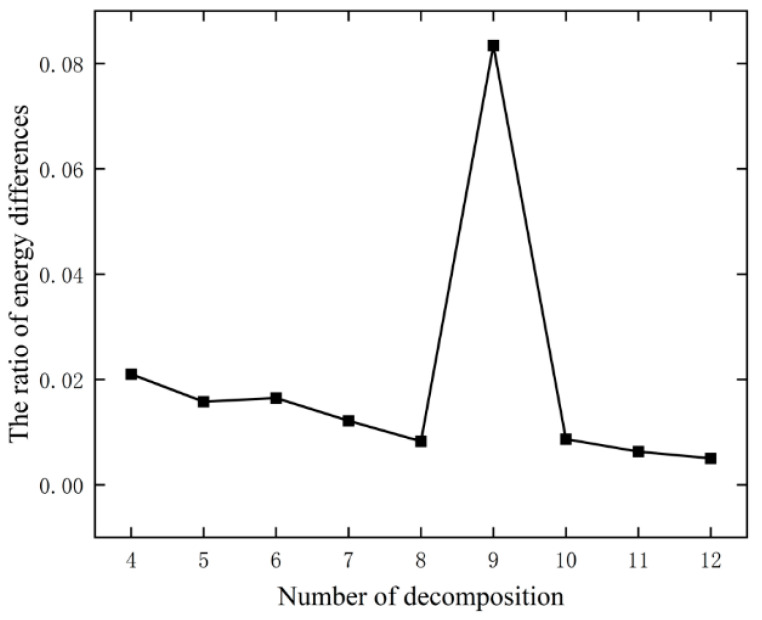
Determination of parameter K using energy difference method.

EMD based on time domain decomposition and VMD based on frequency domain decomposition are used to stabilize the original data. Firstly, decomposition parameter α is pre-set to 300, and then $\theta_{u,u-1}$ under different decomposition numbers is calculated according to Equations (8)–(10) of the energy difference method mentioned above. The results are shown in Figure 6.

**Figure 7:**
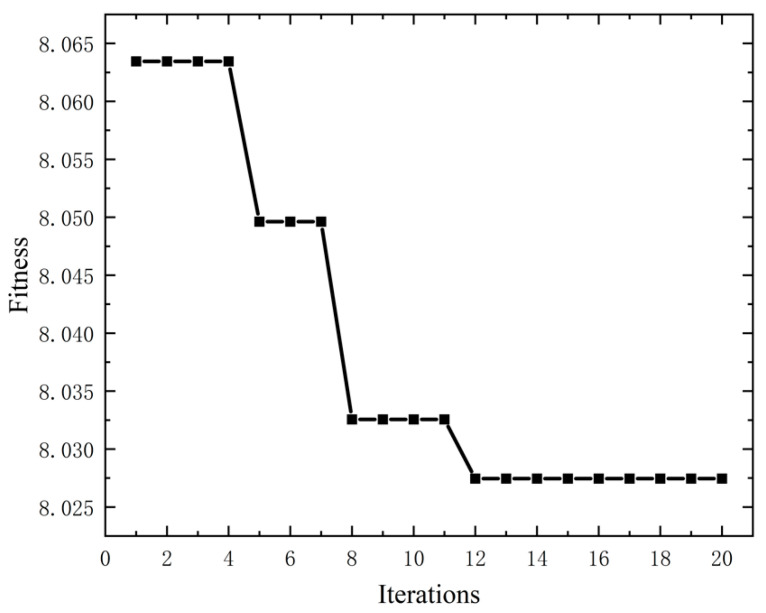
Fitness function diagram of PSO-optimized VMD.

As can be seen from Figure 6, with the increase in decomposition mode K, the ratio of the energy difference mutates at K=9, and the optimal decomposition number is eight. However, this method still requires the artificial setting of parameter α. In order to make up for the shortcomings in the subjective selection, α and K in VMD were optimized using the PSO algorithm with the minimum mean envelopment entropy as the fitness function. The fitness function changes at this time are shown in Figure 7.

**Figure 8:**
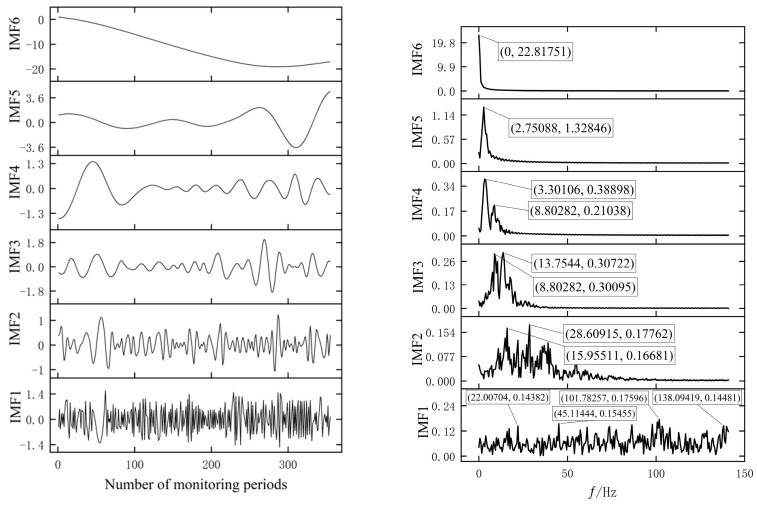
The IMFs decomposed by EMD are shown on the (**left**) and the center frequency corresponding to the IMFs are shown on the (**right**).

**Figure 9:**
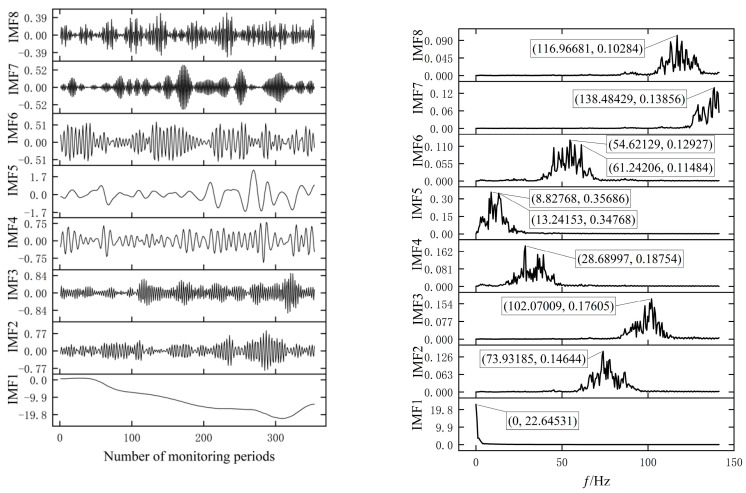
The IMFs decomposed by VMD are shown on the (**left**) and the center frequency corresponding to the IMFs are shown on the (**right**).

In this way, the values of parameter groups α and K of VMD are determined to be [α,K]=[872.079,8.094]. The IMF component and the corresponding spectrum obtained from the original data after processing by the PSO-VMD method and EMD method are shown in Figure 8 and Figure 9.

From Figure 8, it can be seen that the data after EMD decomposition exhibit certain regularity, for example, the IMF1 component obtained after Fast Fourier Transform (FFT) processing has multiple frequency components [25] and the amplitude values (*Y*-axis) of the IMF1 component in Figure 8 are similar at frequencies of 22.00704, 45.11444, 101.78257, and 138.09419 (*X*-axis), indicating the occurrence of a mode-aliasing phenomenon. From Figure 9, it can be seen that the data processed by the VMD method also exhibit good regularity, and the phenomenon of modal aliasing is weakened on different components. As shown in Figure 9, the IMF5 component has similar amplitudes (<0.01) obtained at frequencies of 8.82768 and 13.24153, respectively. Compared with the decomposition results of the EMD method, the mode mixing phenomenon is significantly reduced. This will be beneficial for the prediction model to better capture and learn the characteristics of the data, thereby improving the prediction performance.

### 3.3. Analysis of Model Prediction Effect

All experiments were carried out on computers equipped with AMD R7 cpu and NVIDIA RTX4060 gpu. The model was based on python language, and the highly integrated and modular deep learning framework [26] Tensorflow2.10.0 was adopted to build the model. The learning rate of LSTM and GRU models is 0.005, and the number of iterations is 100.

(1) Experiment 1

In order to clarify the feasibility of the proposed model and the influence of different types of signal processing methods on the model effect, four prediction models were built, respectively, and the deformation values of the next 1 day (Prediction Step = 1) were predicted using the data of each component decomposed above and the raw data without processing, and the time spent for each model prediction was recorded. The average absolute error of the forecast results and the consumption schedule are shown in Table 3.

As can be seen from Table 3, first of all, the four prediction models all show a certain prediction ability for the deformation data. From the prediction error results of the PTB01 monitoring point data, it can be seen that after the data have been processed by VMD, better prediction accuracy can be achieved in the model. At the PTB01 monitoring point, the average absolute error of the PSO-GRU prediction model is 0.127 mm. Compared with the original data and the data decomposed by EMD, the error is reduced by 0.375 mm and 0.335 mm, respectively. At the same time, the PTB04 monitoring point data also showed similar results, and the error was reduced by 0.436 mm and 0.503 mm, respectively. In other words, the deformation data after VMD processing is more conducive to improving the prediction accuracy of the model compared with the original data and EMD method.

Secondly, according to the prediction results of different observation points, the LSTM and GRU models optimized by the PSO algorithm have improved the prediction accuracy compared with the single model. Compared with the single model, the prediction errors of PSO-LSTM decreased by 13.7% and 2.1%, respectively, at different monitoring points, and that of PSO-GRU decreased by 75.9% and 85.2%, respectively, at different monitoring points.

Finally, because the cell structure of GRU is simpler than that of LSTM, it is found that the consumption time of GRU is reduced by 32.1% compared with that of LSTM. However, the consumption of both the LSTM and GRU models optimized by the PSO algorithm is greatly increased because the PSO algorithm consumes a lot of time in the process of hyperparameter optimization, and the prediction time of PSO-GRU is 8.6% shorter than that of PSO-LSTM.

(2) Experiment 2

In order to further verify the effect of the model and the influence of the Prediction step size on the model effect, the data of the PTB01 and PTB04 monitoring points were selected to build four models, respectively, and the deformation values of the next 3 days (Prediction step = 3) and the next 5 days (prediction Step = 5) were predicted. The predicted results are shown in Table 4, and the percentage change of error in the prediction step size from three to five is shown in Table 5.

**Figure 10:**
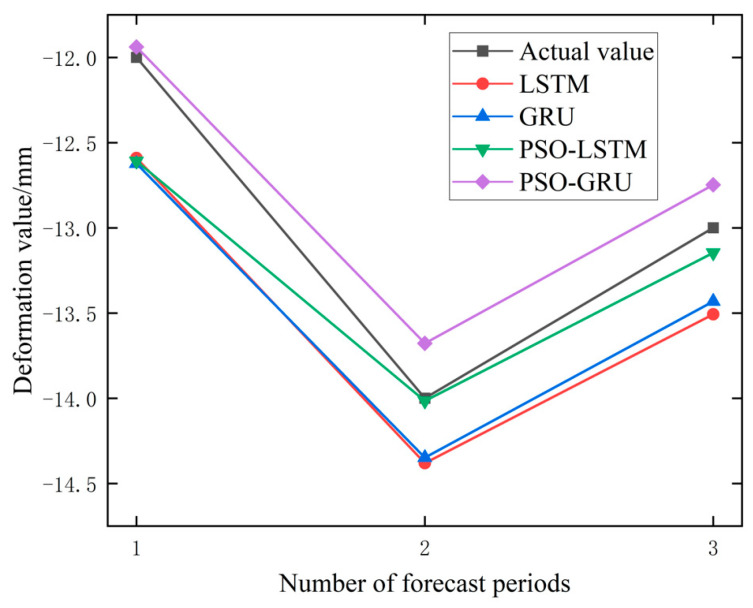
Prediction Results of PTB01 Monitoring Point Data (Step = 3).

**Figure 11:**
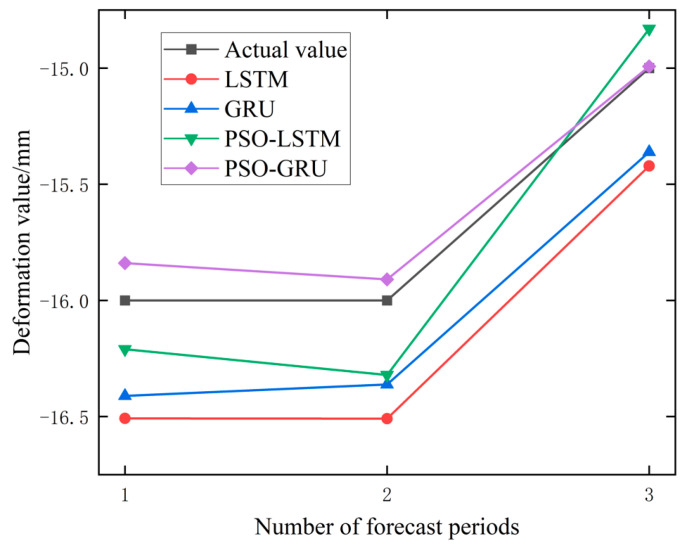
Prediction Results of PTB04 Monitoring Point Data (Step = 3).

**Figure 12:**
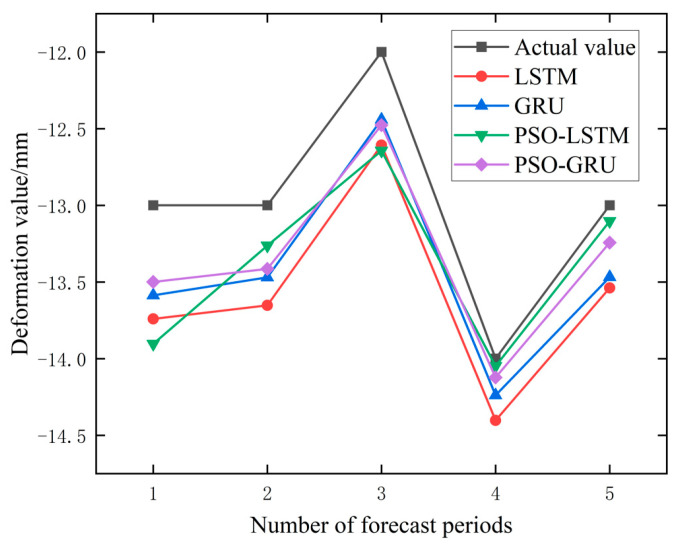
Prediction Results of PTB01 Monitoring Point Data (Step = 5).

**Figure 13:**
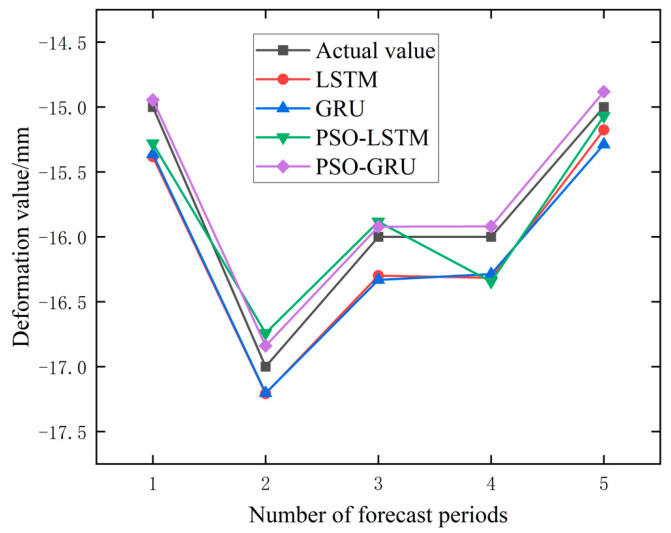
Prediction Results of PTB04 Monitoring Point Data (Step = 5).

It can be seen from Table 4 and Table 5 that the root-mean-square error of some models will also increase when the prediction step size increases. For example, in PTB01, the prediction error of PSO-GRU and PSO-LSTM increases from 0.239 and 0.361 to 0.380 and 0.512, respectively. It is noted that in PTB04, the prediction errors of PSO-GRU and PSO-LSTM decreased from 0.106 and 0.242 to 0.105 and 0.238, respectively, with little variation and basically maintained stability. Overall, the original data, as well as the data processed by the EMD and VMD methods, showed a 28.17%, 3.44%, and 14.24% increase in prediction errors when performing a prediction step of five compared to a prediction step of three. When using raw data for different prediction steps, the prediction error of the prediction model at the PTB01 monitoring point increased by 56.16%, while the prediction error at the PTB04 monitoring point was 0.18%. The percentage increase in the prediction error of the PTB01 data after EMD and VMD processing was −6.53% and 28.76%, respectively. The percentage increase in the prediction error at the PTB04 monitoring point was 13.41% and −0.27%, respectively. Observing the performance of the PSO-GRU data alone, the prediction error increased by 59.00% in the PTB01 data and −0.94% in the PTB04 data. There are also inconsistent changes in the prediction error. In addition, the percentage increase in the error of the four prediction models on three types of data is −7.82%, −17.82%, 54.02%, and 32.75%, respectively. It can be observed that the prediction errors of the PSO-optimized prediction models have all increased to a certain extent, but the percentage increase in the prediction errors of the unoptimized LSTM and GRU prediction models are −7.82% and −17.82%, respectively. The reason for this situation may be that the parameter sets of PSO-LSTM and PSO-GRU are only applicable to the training data when using PTB01 data for training, resulting in a decrease in the generalization ability. The unoptimized parameter settings of LSTM and GRU are not too limited. In summary, from the perspective of the percentage increase in the prediction error, it is believed that an increase in the prediction step size will lead to an increase in the prediction error. The prediction results with prediction steps of three and five are shown in Figure 10, Figure 11, Figure 12 and Figure 13.

In summary, when the original data and the data decomposed by EMD and VMD methods are used for prediction, the data decomposed by VMD are more conducive to the prediction of the model on the same model. LSTM and GRU models optimized by particle swarm optimization are more accurate than those without parameter optimization. Compared with LSTM, the prediction time of GRU is shorter because of its simpler cell structure.

The VMD-GRU deep foundation pit deformation prediction model based on PSO optimization parameters can reduce the error caused by the non-stationarity of original data and the improper selection of hyperparameters, further improve the accuracy of deformation prediction, and verify the feasibility and accuracy of the PSO-VMD-GRU model in deep foundation pit deformation prediction. In addition, compared with the PSO-LSTM deep foundation pit deformation prediction model, PSO-GRU showed better results in terms of the root-mean-square error, average absolute error, and time spent. Meanwhile, similar results were also shown on the deformation data of PTB04 located in the same foundation pit, which verified the superiority of the model. In addition, a comparison of the prediction results of different prediction models proposed in similar articles is summarized in Table 6.

From Table 6, it can be seen that the prediction model proposed in this article has a certain improvement in prediction accuracy (MAE) compared to similar articles (data are taken from the mean absolute error of prediction steps one, three, and five), which can be better applied in engineering.

## 4. Conclusions

The VMD optimized by PSO is used to decompose the non-stationary data of foundation pit deformation, the parameters of GRU are optimized by PSO, and the VMD-GRU deep foundation pit deformation prediction model based on the optimized parameters of PSO is constructed. Eight modal components were obtained by the optimized VMD decomposition. Each component was predicted separately by PSO-GRU, and the final predicted value of foundation pit deformation was obtained by equal weight summation.

Through the analysis of case studies, the results show that the VMD method optimized by PSO is consistent with the results obtained by using the energy difference ratio method in terms of the number of decomposed components. Regarding the PTB01 data decomposed by VMD, compared to the prediction mean square errors of the LSTM, GRU, and PSO-LSTM prediction models, the constructed models reduced their mean square errors by 62.76%, 75.99%, and 53.14%, respectively. Regarding the PTB04 data, it decreased by 70%, 85.17%, and 69.36%, respectively. In addition, compared with the PSO-LSTM model, the model time was reduced by 8.57%. When the prediction step size increased from three to five, the average errors of the four prediction models in the original data, EMD decomposition data, and VMD decomposition data increased by 28.17%, 3.44%, and 14.24%, respectively. The constructed PSO-VMD method can obtain accurate component numbers.

Combining internal and external validation can provide more comprehensive and reliable evaluation results. Based on the above analysis, in terms of internal validation, the traditional K-fold cross validation effect is achieved by dividing the training set, validation set, and test set. In addition, the PTB04 monitoring data are used for the external validation of the constructed model, and the results verify that the model has a good prediction accuracy and certain generalization ability. In addition, by comparing the predictions obtained by different prediction models proposed in different articles, the PSO-GRU prediction model has better prediction accuracy. The increase in the prediction step size will reduce the accuracy of the prediction model to varying degrees, and verify the feasibility and superiority of the prediction model in deep excavation deformation predictions.

In future research, emphasis can be placed on the following aspects:

(1) Due to the relatively singular data of the model constructed, the deformation of deep foundation pit engineering is influenced by multiple factors and it is possible to enhance the dimensionality of the model training data by adding more factor features that affect deformation.
(2) When constructing a network structure, the selection and range of hyperparameters are subjective. Subsequent research can discuss the applicability of network structure parameters in a specific problem through experiments.
(3) In terms of the data used in the prediction model, the impact of different sliding window sizes for time series data on the prediction results can be explored.

## Figures and Tables

**Table 1:** PSO algorithm optimizes the list and range of hyperparameters of GRU.

Parameter	Num Units	Num Layers	Dropout	Epochs	Batch Size
Range	(32, 200)	(2, 5)	(0.0, 0.5)	(5, 25)	(32, 128)

**Table 2:** Raw Data Information Table.

Monitoring Points	Data Acquisition Start Time	Data Acquisition End Time	Number of Samples	Number of Missing Values	Maximum Value	Minimum Value
PTB01	26 May 2022	15 May 2023	280	75	2.00	−23.00
PTB04	26 May 2022	15 May 2023	280	75	2.00	−22.00
PTB08	29 June 2022	15 May 2023	254	67	1.00	−13.00

**Table 3:** Mean absolute error and time consumed of model prediction (Prediction Step = 1).

Prediction Model	PTB01			PTB04			Time Consumed
	Raw Data	EMD	VMD	Raw Data	EMD	VMD	
	(mm)	(mm)	(mm)	(mm)	(mm)	(mm)	(Seconds)
LSTM	1.919	1.729	0.341	1.799	0.729	0.480	25.5
GRU	1.845	2.485	0.529	2.257	0.800	0.971	17.3
PSO-LSTM	0.829	0.794	0.271	0.832	0.672	0.470	882.3
PSO-GRU	0.502	0.462	0.127	0.580	0.647	0.144	806.7

**Table 4:** Root mean square error of model prediction (Prediction Step = 3 and 5).

Prediction Step	Prediction Model	PTB01			PTB04		
		Raw Data	EMD	VMD	Raw Data	EMD	VMD
3	LSTM	0.967	0.653	0.500	1.197	0.883	0.481
	GRU	1.018	0.721	0.482	1.475	0.398	0.379
	PSO-LSTM	0.504	0.849	0.361	0.873	0.663	0.242
	PSO-GRU	0.973	0.753	0.239	0.872	0.269	0.106
5	LSTM	1.025	0.577	0.599	1.058	0.435	0.487
	GRU	0.819	0.490	0.455	1.001	0.327	0.380
	PSO-LSTM	1.703	0.804	0.512	0.975	0.926	0.238
	PSO-GRU	0.976	0.925	0.380	1.158	0.491	0.105

**Table 5:** Percentage Change in Error (Prediction Steps from 3 to 5).

	PTB01			PTB04			PTB01 and PTB04			
Data Type	Raw Data	EMD	VMD	Raw Data	EMD	VMD	Raw Data	EMD	VMD	Mean Value
LSTM	6.00%	−11.64%	19.80%	−11.61%	−50.74%	1.25%	−2.81%	−31.19%	10.52%	−7.82%
GRU	−19.55%	−32.04%	−5.60%	−32.14%	−17.84%	0.26%	−25.84%	−24.94%	−2.67%	−17.82%
PSO-LSTM	237.90%	−5.30%	41.83%	11.68%	39.67%	−1.65%	124.79%	17.18%	20.09%	54.02%
PSO-GRU	0.31%	22.84%	59.00%	32.80%	82.53%	−0.94%	16.55%	52.68%	29.03%	32.75%
Mean value	56.16%	−6.53%	28.76%	0.18%	13.41%	−0.27%	28.17%	3.44%	14.24%	

**Table 6:** Comparison of prediction accuracy (MAE).

Number	Method	Prediction Accuracy (MAE)	Data Sources
1	Grey Wolf Optimization–Extreme learning machine model	0.2614	[27]
2	Auto-Regressive Moving Average Model	0.2006	[28]
3	Back Propagation Neural Network–Auto-Regressive Moving Average Model	0.5762	[29]
4	Auto-regression Model	0.4250	[30]
5	Particle Swarm Optimization–Gate Recurrent Unit	0.1486 (PTB04)	This paper
6		0.3336 (PTB01)	

## Data Availability

Data are contained within the article.

## Nomenclature

Abbreviations	
PSO	Particle Swarm Optimization
VMD	Variational Mode Decomposition
GRU	Gated Cycle Unit
LSTM	Long Short-Term Memory
IMF	Internet Message Format
Symbols	
*f*(*t*)	Monitoring data sequence
*α*	Penalty parameter
*K*	Number of decompositions
*y*	Interpolated data
*Y*	Predictive value
*θ* *_u,u_*_−1_	Energy difference ratio
*m*	Prediction step
*Si*	Particle Position
*Vi*	Particle Velocity
*Pi*	Individual Extremum
*G*	Overall Extremum
*E_mse_*	Mean Square Error
*E_mae_*	Mean Absolute Error

## References

[1] Yao Y., Becker J.M., Ford M.R., Merrifield M.A. (2016). Modeling wave processes over fringing reefs with an excavation pit. Coast. Eng..

[2] Luo X.S., Qing N.R. (2018). Displacement prediction of Bai jia bao landslide based on empirical mode decomposition and long short-term memory neural network in Three Gorges area, China. Comput. Geosci..

[3] Zhang S., Tan Y. (2022). Deformation prediction of foundation pit based on long short-term memory algorithm. Tunn. Constr..

[4] Hochreiter S., Schmidhuber J. (1997). Long short-term memory. Neural Comput..

[5] Hu Y., Luo D., Hua K., Zhang X. (2019). Overview on deep learning. CAAI Trans. Intell. Syst..

[6] Cho K., Van Merriënboer B., Gulcehre C., Bahdanau D., Bougares F., Schwenk H., Bengio Y. (2014). Learning phrase representations using RNN encoder-decoder for statistical machine translation. Proceedings of the 19th Conference on Empirical Methods in Natural Language Processing (EMNLP).

[7] Sepehri M., Apel D.B., Hall R.A. (2017). Prediction of mininginduced surface subsidence and ground movements at a Canadian diamond mine using an elastoplastic finite element model. Int. J. Rock Mech. Min. Sci..

[8] Luo X., Gan W., Wang L., Chen Y., Meng X. (2020). A Prediction Model of Structural Settlement Based on EMD-SVR-WNN. Adv. Civ. Eng..

[9] Yang S., Yang H., Li N., Ding Z. (2023). Short-Term Prediction of 80–88 km Wind Speed in Near SpaceBased on VMD–PSO–LSTM. Atmosphere.

[10] Liang D., Xu J., Li S., Sun C. Short-term passenger flow prediction of rail transit based on VMD-LSTM neural network combination model. Proceedings of the 2020 Chinese Control And Decision Conference (CCDC).

[11] Li H., Zhao Z., Du X. (2022). Research and Application of Deformation Prediction Model for Deep Foundation Pit Based on LSTM. Wirel. Commun. Mob. Comput..

[12] Cui D., Zhu C., Li Q., Huang Q., Luo Q. (2021). Research on Deformation Prediction of Foundation Pit Based on PSO-GM-BP Model. Adv. Civ. Eng..

[13] Liu Q., Yang C.Y., Lin L. (2021). Deformation Prediction of a Deep Foundation Pit Based on the Combination Model of Wavelet Transform and Gray BP Neural Network. Math. Probl. Eng. Theory Methods Appl..

[14] Jing C., Wang H., Li H. (2021). Deformation Prediction of Foundation Pit Based on Exponential Power Product Model of Improved Algorithm. Geofluids.

[15] Ma Q., Liu S., Fan X., Chai C., Wang Y., Yang K. (2020). A Time Series Prediction Model of Foundation Pit Deformation Based on Empirical Wavelet Transform and NARX Network. Mathematics.

[16] Shi Y.H., Eberhart R.C. (2020). Empirical study of particle swarm optimization. Proceedings of the Congress on Evolutionary Computation.

[17] Poli R., Kennedy J., Blackwell T. (2007). Particle swarm optimization. Swarm Intell..

[18] Dragomiretskiy K., Zosso D. (2014). Variational modedecomposition. IEEE Trans. Signal Process..

[19] Zhou F., Yang X., Shen J., Liu W. (2020). Fault Diagnosis of Hydraulic Pumps Using PSO-VMD and Refined Composite Multiscale Fluctuation Dispersion Entropy. Shock Vib..

[20] Zhang Y., Li R., Zhang J. (2021). Optimization scheme of wind energy prediction based on artificial intelligence. Environ. Sci. Pollut. Res. Int..

[21] Wang X.-B., Yang Z.-X., Yan X.-A. (2017). Novel Particle Swarm Optimization-Based Variational Mode Decomposition Method for the Fault Diagnosis of Complex Rotating Machinery. IEEE/ASME Trans. Mechatron..

[22] Yang L., Wu Y., Wang J., Liu Y. (2018). Research on recurrent neural network. J. Comput. Appl..

[23] Le Q.V., Jaitly N., Hinton G.E. (2015). A simple way to initialize recurrent networks of rectified linear units. arXiv.

[24] Wang J., Cao J., Yuan S., Cheng M. (2021). Short-term forecasting of natural gas prices by using a novel hybrid method based on a combination of the CEEMDAN-SE-and the PSO-ALS-optimized GRU network. Energy.

[25] Zhang W., Wang T. (2022). Short-term power load forecasting model design based on EMD-PSO-GRU. Sci. Program..

[26] Kiran T. (2020). Computer vision accuracy analysis with deep learning model using Tensor Flow. Int. J. Innov. Res. Comput. Sci. Technol..

[27] Yang S., Yang Z., Zhang L., Guo Y., Wang J., Huang J. (2023). Research on deformation prediction of deep foundation pit excavation based on GWO-ELM model. Electron. Res. Arch..

[28] Guanming G. (2020). Research on Deformation Prediction of Deep Foundation Pits Based on Gene Expression Programming. Proceedings of the 2020 International Conference on Urban Engineering and Management Science (ICUEMS).

[29] Hu Y.B., Shao F., Huang Y.X., Liu Y.W., Liang J.J. (2015). Application of BP-ARMA Combined Model based on Entropy Method in the Prediction of Circle Beam Displacement of Foundation Pit. Appl. Mech. Mater..

[30] Tian L., Hua X.S. (2007). Settlement prediction for buildings surrounding foundation pits based on a stationary auto-regression model. J. China Univ. Min. Technol..

